# The Association of Education, Employment and Living with a Partner with the Treatment among Patients with Head and Neck Cancer

**DOI:** 10.3934/publichealth.2015.1.1

**Published:** 2015-01-23

**Authors:** Gabriela Štefková, Zuzana Dankulincová Veselská, Viola Vargová, Marek Paľo

**Affiliations:** 1 Nursing Department, Medical Faculty, PJ Safarik University in Košice, Slovakia; 2 Institute of Public Health, Medical Faculty, PJ Safarik University in Košice, Slovakia; 3 1st Department of Internal Medicine, Medical Faculty, PJ Safarik University in Košice, Slovakia; 4 Department of Radiology of JA Reiman University hospital in Prešov, Slovakia

**Keywords:** head and neck cancer, social status, socioeconomic status, treatment

## Abstract

The aim of this study was to explore possible associations between social and socioeconomic status and ongoing treatment among patients with head and neck cancer.

**Material and methods:**

Data from 159 examined patients treated with head and neck cancer during the period from 2011 to 2012 were explored. A logistic regression analysis was used to assess association of social status (living with somebody vs. living alone), socioeconomic status (employed vs. unemployed) and education (primary/secondary/university) with treatment.

**Results:**

The results from logistic regression showed significant association of employment status and education with both interruption in radiochemotherapy and searching for additional help after surgery. Interruption of radiochemotherapy was almost 3 times more likely in a group of unemployed compared to the employed patients. Lack of searching for help after surgery was almost 4 times more likely in a group of unemployed compared to the employed and 5 times more likely in the group with the lowest education compared with the group with the highest education.

**Conclusions:**

The study suggests that special attention needs to be paid, not only during but also after treatment, to the patients from low socioeconomic groups.

## 1. Introduction

Improved diagnosis and treatment, increasing incidence rates and a prolonged life expectancy have steadily increased the number of people living with a cancer diagnosis [1]–[3]. Head and neck cancer is one of the sixth most prevalent worldwide neoplasms [4]–[7] independently of tumour site (oral cavity, oropharynx, larynx and hypopharynx). The cancer of oral cavity is difficult to treat due to anatomic site. Patients experience a deterioration of their basic functions affecting such important areas as breathing, mastication, salivating, swallowing, speaking, senses (hearing, taste andsmell) [8]. The treatment of patients with head and neck cancer depends on a number of factors, including the exact location of the tumour, the stage of the cancer, and the person's age and general health. Usually treatment of patients can include surgery, radiation therapy, chemotherapy, targeted therapy, or a combination of treatments. Head and neck surgery need a multidisciplinary approach before, during, and after cancer treatment. A multidisciplinary oncology team that includes oncologists, oncology nurses, and dental generalists and specialists as well as dental hygienists, social workers, dieticians, and related health professionals can often achieve highly effective preventive and therapeutic outcomes relative to oral complications in these patients [9].

Cancer of the head and neck may long remain without symptoms or is diagnosed in the early stages as minor chronic inflammatory disease [10]. The disease is more common in men than women in most countries. The risk of developing disease increases with age in patients over age 50. However, oral cancers are relatively common in young and middle aged among men and women [11]. In 2012 in Slovak Republic an estimated 24 045 newly cases of cancer have been diagnosed, and 11 783 people die from the disease. Annually about 300 patients are being diagnosed with cancer of larynx specifically. More than 90% of those patients are men and more than 98% of patients are smokers. They are usually from lower social groups without health awareness [12],[13].

Management of patients with head and neck cancer therapy includes identification of high-risk factors (e.g. smoking, alcohol use), patient education, social status, and early initiation of interventions [14]. Any diagnosis of cancer carries emotional, social, and physical challenges [15] and may trigger potential socioeconomically problems for patients [16],[17]. The process of treatment often causes inability to be fully engaged in the work market [18], [19]. There are complex interactions between social problems and cancer which are an underlying reflection of the life, social status or socioeconomic status of the individual patients [20]. Routine assessment of social problems and socioeconomic status is not part of standard oncology practice [21]. At the same time people from deprived groups are the most likely to delay seeking medical advice [17],[22].

Achieve good health in cancer patients with lower social support and socioeconomic status is a challenging public health concern. The aim of this study was to explore possible associations between social and socioeconomic status and ongoing treatment among patients with head and neck cancer.

## 2. Methods

### 2.1. Sample

One hundred and fifty nine adult patients with at all stages of head and neck cancer were enrolled in the cross-sectional study during the period from 2011 to 2012 from the Department of Radiation Oncology of JA Reiman University hospital in Prešov. A multi-disciplinary team at the Department of Radiation Oncology is made up of surgical, medical, and radiation oncologists, as well as social workers and nurses when treating cancer. The goal of department is to provide comprehensive care tailored to patient needs—with the ultimate result of eradicating cancer and relieving symptoms. The study included patients treated within outpatient treatment and the patients at the Department of Radiation Oncology, who voluntarily agreed to participate in the study. The results of study represent eastern region of Slovakia as the catchment area for JA Reiman University hospital in Prešov. All of the approached patients agreed to participate in the study and response rate was 100%. Data were obtained from questionnaires and medical files of outpatient and inpatients. Respondents belonged to age groups which ranged from 31 to 75 years. The most frequent age group consisted of patients from 45 and 59 years of ages (64.6% out of 159 patients). Gender of patients with head and neck cancer were mostly males (96.2%) in comparison with females (3.8%). All patients signed an informed consent statement before interview. Participation on study was voluntary, and ethical committee approved this study.

### 2.2. Measures

Demographic measures were explored using single item questions about age and gender. Age was retrieved from the medical files. Data regarding education, employment, living with or without partner, interruption of treatment and searching for additional help after surgery was obtained from questionnaires.

Highest achieved education was measured with three possible response categories: “primary education”, “secondary education”, and “higher education”.

Employment status was measured by question “At this time you are” with six possible response categories: “employed”, “employed with half disability”, “self-employed”, “unemployed”, “disability pension”, “pensioner”. Employment status was dichotomized into two groups employed and unemployed. Employed group include patients: “employed”, “employed with half disability”, “self-employed”. Unemployed group include patients: “unemployed”, “disability pension”, “pensioner”.

Social status was measured by question “At this time you are living”: with five possible response categories: “living alone”, “living with wife”, “living with wife and children”, “living with parents”, “living with friends”. Social status was dichotomized into two groups as living with somebody and living alone. Group of patients living with somebody include: “living with wife”, “living with wife and children”, “living with parents”, “living with friends”.

Interruption of treatment during therapy was measured by question “Have you interrupted therapy during treatment?” with three possible response categories: “without interruption”, “once”, “two times and more”.

Searching for additional help after surgery was measured by question “After surgery you were asking for additional help (with exception of your GP, ORL and oncology doctor)” with eight possible response categories: “psychologist”, “family and friends”, “social worker”, “nursing home care”, “priest”, “internet”, “civil association”, and “none”. Searching for additional help after surgery was dichotomized into two groups: “none” versus all other categories. Categories about social status and searching for help were created for this research as measure of impact of management on treatment among cancer patients.

### 2.3. Statistical Analysis

We used basic descriptive statistics in the first step. Next, logistic regression was performed to explore the association between socioeconomic status, social status, and education as independent variables and interruption of treatment and searching for help after surgery as dependent variables. All analyses were done using IBM SPSS Statistics 20.

## 3. Results

Characteristics of the patients participating in the study are shown in Table 1. Of the total group of patients with the head and neck cancer 76.10% did not interrupt their radiochemotherapy and 23.90% of patients interrupt therapy at least once. From all the patients only 35.7% searched for help, 64.3% did not search for additional help after surgery.

**Table 1.** Descriptive statistics (n, %) of the studied variables (N = 159).

**Gender n (%)**	
male	153 (96.2)
female	6 (3.8)
**Age groups n (%)**	
31–44	5 (3.2)
45–59	102 (64.6)
60–74	50 (31.6)
75 and more	1 (0.6)
**Education n (%)**	
primary	49 (31.2)
secondary	94 (59.9)
higher	14 (8.9)
**Employment status n (%)**	
employed	50 (31.4)
unemployed	109 (68.6)
**Social status n (%)**	
living with somebody	108 (67.9)
living alone	51 (31.4)
**Interruption of treatment n (%)**	
uninterrupted	121 (76.1)
interrupted at least once	38 (23.9)
**Searching for additional help after surgery n (%)**	
yes	55 (35.7)
no	99 (64.3)

Table 2 shows association of education, employment status and social status on interruption of radiochemotherapy between cancer patients. Logistic regression (OR and 95% CI) confirmed the significant association between socioeconomic status (employed vs unemployed), and interruption of radiochemotherapy. Interruption of radiochemotherapy was almost 3 times more likely in a group of unemployed compared to the employed patients.

**Table 2.** Association of education, employment and social status with interruption of radiochemotherapy based on logistic regresion (OR a 95% CI) mutually adjusted.

		OR (95% CI)mutually adjusted
Education	Higher	Ref
	Secondary	3.42 (0.39–29.71)
	Primary	3.81 (0.46–31.90)
Employment status	Employed	Ref
	Unemployed	2.78 (1.05–7.31)*
Social status	Living with somebody	Ref
	Living alone	0.79 (0.55–2.92)

Ref. = reference category, *p* < 0.05*

Approximately only one third of patients (35.7%) searched for additional help after discharge to home care after surgery. Table 3 shows association of education, employment status and social status on searching for additional help after discharge to home care after surgery. Logistic regression (OR and 95% CI) confirmed the significant association between socioeconomic status (employed vs. unemployed) and education (primary vs. university) and searching for additional help after surgery. Lack of searching for help after surgery was almost 4 times more likely in a group of unemployed compared to the employed and 5 times more likely in the group with the lowest education compared with the group with the highest education.

**Table 3.** Association of education, employment and social status with searching for help after surgery based on logistic regresion (OR a 95% CI) mutually adjusted.

		OR (95% CI)
Education	University	Ref
	Secondary	3.64 (0.97–13.69)
	Primary	4.54 (1.23–18.23)*
Employment status	Employed	Ref
	Unemployed	3.90 (1.83–8.29)***
Social status	Living with somebody	Ref
	Living alone	1.49 (0.65–3.39)

Ref. = reference category, *p* < 0.05* *p* < 0.001***

## 4. Discussion

The aim of this study was to explore possible associations between social and socioeconomic status and ongoing treatment among patients with head and neck cancer and describes influence of social and socioeconomic status among head and neck cancer patients. Logistic regression confirmed the significant association of employment status (employed vs. unemployed), with the interruption of radiochemotherapy and association of employment status (employed vs. unemployed) and education (primary vs. university) with searching for additional help after surgery. Interruption of radiochemotherapy was almost 3 times more likely in a group of unemployed compared to the employed patients. Lack of searching for help after surgery was almost 4 times more likely in a group of unemployed compared to the employed and 5 times more likely in the group with the lowest education compared with the group with the highest education. More than half of patients with cancer was at time of diagnosis unemployed, and of these patients confirmed 3 times more likely interruption of radiochemotherapy, and 4 times more likely lack of searching for help after surgery compared to the employed patients.

Cancer is a serious disease that brings a series of changes not only for the health status of patients, but significantly interferes also with their work and professional life as cancer patients, are often forced to change jobs or stop working at all [18].

Diagnosis of cancer carries along modification of health and significantly interferes with family, work and personal life of people. Radiation therapy represents the first line of treatment for most cancers. Cancer treatment is not complete without a care for the patient after treatment. The management of treatment is often disturbed of cancer patients from the lower social groups [9].

Several authors observed correlation between education and cancer treatment [23]. Lower education level may impair the ability of an individual to use available healthcare services for prevention, clinical procedures, and follow-up [24],[25]. In addition, several studies reported that socio-economic status is also an important prognostic factor among most common cancers patients [20],[26] and indicate survival differences among patients from various socioeconomic groups [27]. Insufficient education is also considered a risk factor for a poor prognosis, delayed diagnosis and an incorrect choice of treatments [24],[28]. Lack of searching help after surgery of patients with lowest education was 5 times more likely for compared with the group with the highest education. Failure to seek additional help is however not necessarily a negative sign, but it might be regarded in a positive way. For example, those patients who do not seek additional help may not do so because their performance status is sufficient and they do not need it. Or they may be independent in their nature and not used to seeking for help.

The Slovak Republic is among the EU countries with the highest levels of unemployment between young people and women particularly hard-hit. The percentage of the labour force that has been unemployed for a year or longer is currently at nearly 8.9%. Employment rates are generally higher for individuals with a higher level of education; in the Slovak Republic, an estimated 77% of individuals with at least a tertiary education have a paid job, compared with an estimated 15% for those without an upper secondary education, around 60% of the working-age population aged 15 to 64 has a paid job. Current situation about education in Slovak republic includes 68,6% citizens of adults aged 25–64 have of secondary degree, around 16,6% primary degree and 12,6% tertiary stages of education [29].

Defining social problems, and economic problems are difficult but the range of potential problems is enormous. Oncology therapy is not clear without care of patients after surgery. However, the detection and characterisation of social problems may lead to an improvement in the care of cancer patients and result in enhanced patient well-being. Cancer patients must be under constant medical supervision. Researchers have also suggested that support from family members, or support group (e.g. psychologist, civil association) may play an important role in the adjustment of cancer patients [15],[30] through sharing the burden of treatment.

Insufficient social support is a risk factor for a poor prognosis of diagnosis and an incorrect choice of treatments [28]. Previous studies confirmed that married individuals have significantly better survival [20] than widowed or non-married subjects [31]. We can expect that social support from family is usually obligatory, but from friends can be voluntary [15],[32]. However, our results did not confirm the impact of social status on the course of treatment and care of patients with head and neck cancer, unlike above mentioned a study, which highlights the significant impact of social support in cancer and chronically ill patients. The reason for the different findings in our study may be the use of a single question instead standardized scale for social support.

Establishing follow-up of cancer patients with lower socioeconomic status after discharge should be a priority of hospital-based physicians caring for such patients. People with cancer disease get to prompt appointments and telephone contact; for providing emotional and family support; and for referral, triage, and general medical care [33]. It is suggested for hospitals to provide available peer counselling programs for patients in need.

We are aware that small sample in our study leads to limited generalizability and it need to be explored on the larger samples in the future research. First of all, rather small sample size and patients being enrolled from only one hospital is making generalization of our findings problematic and should be repeated on the larger, more nationally representative sample. In order to cover chosen aspects of socioeconomic and social status only basic variables were used and those should be once again extended in future research. Last but not least, cross-sectional design of the study with self-reported answers given through questionnaires creates additional limitation, however provide initial overview of the topic and suggest direction for possible future research. Another limitation is use of self-reported questions which might influence our findings to some extent.

## 5. Conclusion

This study was focused primarily on the impact of social and socioeconomic status on the treatment among cancer patients. The short-term and intermediate effects of head and neck cancer and its treatment are well documented [34]. Despite the availability of good health care, there is more important factor for health care patients (lower socioeconomic status, lower education, interruption therapy and searching for additional help after surgery among patients with head and neck cancer). Treatment itself and especially in convalescent phases might be even more complicated by attitude to life oriented on the presence and in the cancer patients with minimal focus on the future. Our results underline the need for educating oncologists in order to improve their ability to identify those patients with more demanding socioeconomic position. The identification of potential problems may lead to an improvement in the care of cancer patients and result in enhanced patient well-being whether it is through providing emotional and family support, counselling by a social worker, or by improvement of general medical care [33]. Likewise, public health programs aimed at increasing preventive behaviours among low-income people may also lessen the gap in cancer outcomes [35]. It is important to find out whether socio-economic differences in how patients seek and obtain access to health services, or participate in screening, are associated with socio-economic differences in cancer survival [20].
